# In Vitro Antiprotozoal Activity of *Hibiscus sabdariffa* Extract against a Ciliate Causing High Mortalities in Turbot Aquaculture

**DOI:** 10.3390/biology12070912

**Published:** 2023-06-26

**Authors:** Ana Carvalho, Inês Domingues, Carla Carvalho, Artur M. S. Silva, Amadeu M. V. M. Soares, Catarina R. Marques

**Affiliations:** 1 Centre for Environmental and Marine Studies (CESAM), Department of Biology, University of Aveiro, Santiago University Campus, 3810-193 Aveiro, Portugal; 2 Laboratório Associado para a Química Verde (LAQV)—REQUIMTE, Department of Chemistry, University of Aveiro, Santiago University Campus, 3810-193 Aveiro, Portugal

**Keywords:** *Philasterides dicentrarchi*, natural products, population growth, oxidative stress, neurotoxicity, proteases activity, protease gene expression, UHPLC-ESI-MS

## Abstract

**Simple Summary:**

The in vitro antiprotozoal activity of *Hibiscus sabdariffa* ethanolic extract was evaluated on the ciliate parasite *Philasterides dicentrarchi*, which causes severe losses in turbot aquaculture. Our results showed that *H. sabdariffa* extract had successfully inhibited the parasite population growth rate (IC_50_ = 1.57 mg mL^−1^), and caused significant changes in the activity of antioxidant enzymes (LOEC = 0.22 mg mL^−1^), especially glutathione peroxidase, total glutathione, and catalase. The activity of proteases (virulence factors) was also inhibited (IC_50_ = 0.76 mg mL^−1^), and the gene expression of catepsin 90 and leishmanolysin proteases was downregulated. Organic acids and phenolic phytochemicals in hibiscus extract are potentially responsible for the antiprotozoal bioactivity herein determined. Therefore, *H. sabdariffa* extract can be a promising disease-control alternative against the ciliate proliferation, cellular defense mechanisms, and pathogenicity.

**Abstract:**

*Philasterides dicentrarchi* is an histophagous parasite that infects flatfish, namely turbot (*Scophthalmus maximus*), and cause significant losses in aquaculture units. The available measures for *P. dicentrarchi* control have limited efficiency, and some cause harm to fish. Hence, sustainable and natural control strategies are urgently needed. This study evaluated the in vitro bioactivity of the ethanol extract of *Hibiscus sabdariffa* calyces on *P. dicentrarchi* population growth rate (PGR), oxidative stress biomarkers (glutathione-*S*-transferases (GST), glutathione reductase (GR), glutathione peroxidase (GPx), total glutathione (TG) and catalase (CAT), neurotoxicity (acetylcholinesterase, AChE), activity and gene expression of proteases as major virulence factors. *H. sabdariffa* extract inhibited parasite PGR (IC_50_ = 1.57 mg mL^−1^), and caused significant changes in the activity of antioxidant enzymes (LOEC = 0.22 mg mL^−1^), especially GPx, TG, and CAT. The activity of proteases was also severely inhibited (IC_50_ = 0.76 mg mL^−1^), and gene expression of catepsin 90 and leishmanolysin proteases was downregulated. Organic acids and phenolic phytochemicals in hibiscus extract are potentially responsible for the antiprotozoal bioactivity herein determined. Therefore, *H. sabdariffa* extract can be a promising disease-control alternative against the ciliate proliferation, cellular defense mechanisms and pathogenicity. Still, its applicability in aquaculture settings, and potential effects on farmed fish, should be further elucidated.

## 1. Introduction

*Philasterides dicentrarchi* (subclass Scuticociliatia, phylum Ciliophora) are originally free-living protozoa, which can become opportunistic histophagous parasites capable of causing severe infections and mortality outbreaks in several fish species (e.g., *Dicentrarchus labrax*, *Paralichthys olivaceus*) [1]. In particular, turbot (*Scophthalmus maximus*) produced in intensive aquaculture units [2] has been often affected with the disease provoked by this parasite (i.e., scuticociliatosis), that results in great economic and production losses [1].

Despite the critical constraints frequently caused, the mechanisms of *P. dicentrarchi* infection and pathogenicity have not been so far fully elucidated. Notwithstanding, there are evidences that a potential infection pathway may be associated with the synthesis and release of proteases by the ciliates [3,4]. Such proteases act as virulence factors by promoting the digestion of fish tissues, modulating the function of leukocytes [5], and contributing to overall evasion from the fish immune response [6,7]. Cysteine proteases are one type of proteases likely involved in parasite infectivity, being thereby considered a potential target for the treatment of scuticociliatosis [3,5]. Leishmanolysin proteases are also critical in host invasion and immune system modulation [7], being upregulated in *P. dicentrarchi*-infected turbots [8]. Besides the proteases, *P. dicentrarchi* can benefit from a robust antioxidant system that boosts its resilience to reactive oxygen species (ROS) produced along host’s infection and immune response. Folgueira et al. [9] observed that the generation of toxic products, such as ROS, triggered the activity of antioxidant enzymes in *P. dicentrarchi* (e.g., superoxide dismutase, catalase, glutathione peroxidase) to ensure the parasites’ survival. Therefore, the analysis of ROS production in *P. dicentrarchi* can be exploited as a biomarker of oxidative stress and also as a target for controlling the proliferation of the parasite.

Since the appearance of scuticociliatosis events in aquaculture units, several mitigation measures of *P. dicentrarchi* proliferation have been attempted [10]. Nevertheless, none have so far resulted in an effective control or eradication of the parasite, mainly due to its high virulence, genetic variability and endoparasitic behaviour [4]. Some substances previously tested, such as oxyclozanide and niclosamide, demonstrated to be effective against *P. dicentrarchi*, but they were toxic to zebrafish [10]; while formalin, which is regularly applied, is a known carcinogen [11]. *P. dicentrarchi* showed to be sensitive to several antiprotozoal drugs in vitro. However, these substances did not successfully treat systemic infections [2], besides contributing for the environmental burden regarding the widespread of antimicrobial resistance. Efforts have been devoted to the development of vaccines against scuticociliatosis through the stimulation of the fish immune system. Although the created vaccines protected turbot against homologous isolates of *P. dicentrarchi*, this protection becomes low or null against other non-homologous isolates or serotypes, which regularly co-occur in aquaculture units [2].

Currently, the aquaculture is globally seeking for sustainable and environmental-friendly disease management schemes and products, that may help reaching the United Nations Sustainable Development Goals established for aquaculture. In this context, nature-based solutions have been preferably claimed, in which plants can be excellent candidates, since they are a source of (secondary) metabolites with biological activity. These metabolites have a key role in defense mechanisms against phytopathogens (microbes) and predators [12]. Medicinal plants have already been used as feed additives and antimicrobials in aquaculture, and were shown to stimulate fish growth performance, appetite, and digestive activity, as well as to reduce stress and improve the immune system activity [13].

*Hibiscus sabdariffa*, commonly known as roselle or hibiscus, is an herbaceous shrub of the Malvaceae family used in traditional gastronomy, as well as for medicinal purposes [14]. The medicinal potential of *H. sabdariffa* has been associated with the abundance of bioactive substances [12,15], such as tannins, phenols, and flavonoids, among which flavonols and anthocyanins are remarkable for its antioxidant and antibacterial properties [16,17]. Indeed, several studies determined the antimicrobial activity of *H. sabdariffa* extracts against Gram-negative (e.g., *Escherichia coli*, *Pseudomonas aeruginosa*, *Klebsiella pneumoniae*, *Salmonella typhi*, *Salmonella enteritidis*) and Gram-positive bacteria (e.g., *Bacillus subtilis*, *Staphylococcus aureus*, *Micrococcus luteus*), fungi (e.g., *Candida albicans*), and in lesser extent, protozoans (clinical isolate *Giardia lamblia*) [12,15,18,19,20]. Nevertheless, the antiprotozoal activity of *H. sabdariffa* has not yet been tested against this parasite or other ciliates.

Hence, we hypothesized that *H. sabdariffa* may contain extractable chemical compounds with bioactive nature, capable of impairing the proliferation, antioxidant balance, and infectivity of the parasite. Therefore, the goal of the present work was to evaluate the in vitro effect of *H. sabdariffa* ethanol extract on the population growth rate, oxidative stress responses, proteases activity and differential expression of relevant protease-encoding genes of *P. dicentrarchi*. In parallel, a qualitative chemical analysis of the crude extract was performed by UHPLC-ESI-MS to find out the potential presence of bioactive compounds. Our major finding was that the *H. sabdariffa* crude extract exerted an antiprotozoal activity that may be associated with the presence of certain organic acids and polyphenolic compounds. More specifically, it was observed the impairment of biological responses of *P. dicentrarchi*, which are pivotal to the physiological balance, virulence and survival of the fish parasite, thereby reinforcing that *H. sabdariffa* has promising properties to be exploited in the future for mitigating the proliferation of this ciliate in turbot aquaculture.

## 2. Materials and Methods

### 2.1. Isolation and Culture of Parasites

*P. dicentrarchi* individuals were isolated from infected turbots in a local Portuguese aquaculture facility, as previously described by Iglesias et al. [21]. The parasites were cultured in the laboratory using T-25 cell culture flasks containing Leibovitz’s L-15 medium supplemented with FBS (10%) and antibiotic-antimycotic solution (10%) [21]. The cultures were kept at 20 °C ± 1 °C during 4 days, being then sub-cultured into fresh medium.

### 2.2. Preparation of H. sabdariffa Extract

Dried calyces of *H. sabdariffa* originally cultured in Egypt were obtained in a local provider (ADP, Lda., Aveiro, Portugal). The calyces were mechanically grounded using a Bosch MKM6000 grinder (Aveiro, Portugal). The powder was immersed in ethanol 96% (1:10 powder:ethanol) and left for 24 h in an orbital shaker at 125 rpm and 25 °C. The resulting mixture was centrifuged at 4000 rpm for 15 min at 4 °C, preserving the supernatant, which was afterwards filtered through qualitative filter paper grade 1. The ethanol was evaporated in a Heidolph Hei-VAP Core rotary evaporator (Schwabach, Germany), at 86 rpm, employing 90 mbar pressure, with a bath temperature of 41 °C. The resulting crude extract was weighed, dissolved in acetone (according to the stock concentration of crude extraction solution prepared), and stored in the dark at 4 °C until further processing.

### 2.3. Analysis of H. sabdariffa Extract Bioactivity

#### 2.3.1. Effect on Parasite Population Growth

*P. dicentrarchi* was exposed to a range of sub-lethal concentrations of *H. sabdariffa* crude extract (0.04–2.00 mg mL^−1^), which were prepared by dilution with culture medium from a stock solution of 1 g mL^−1^. Three replicates were considered *per* test concentration, plus the negative control (no added extract) and solvent control (added with the maximum concentration of acetone, i.e., 100 μL mL^−1^). The exposures were performed in 24-well plates (initial cell density of 1.5 × 10^4^ cells mL^−1^), during 96 h (i.e., 4 days), at a temperature of 20 °C ± 1 °C. At the end, *P. dicentrarchi* cells in each well were fixed with glutaraldehyde, and the number of viable cells were counted in a Neubauer chamber. The population growth was calculated from the determined cell density (cells mL^−1^), following Equation (1).

Growth rate = (Log(CD_96_) − Log(CD_0_))/Δt (1)

where CD_96_ represents the ciliate cell density at 96 h, and CD_0_ represents the cell density at 0 h of exposure, while Δt represents the duration of the experiment (4 days). The growth rate was subsequently expressed as a percentage of the negative control. The parasites in the microplate wells were microscopically observed in an inverted microscope (Axio Vert.A1, Zeiss, Jena, Germany) at the beginning and end of the assay.

#### 2.3.2. Oxidative Stress Biomarkers

*P. dicentrarchi* was exposed to a range of concentrations of the plant extract (0.14–1.94 mg mL^−1^) in 24-well plates, for 2 h, at a temperature of 20 °C ± 1 °C, for biomarker analyses. The number of replicates per concentration and controls (negative and solvent controls) was 9. At the end of the exposure, the cells were removed from the wells, washed twice in phosphate buffer saline 1×, and resuspended in potassium phosphate buffer (0.1 M, pH 7.4). Cell suspensions were homogenized using an ultrasonic homogenizer (Branson Ultrasonics Sonifier S-250A, Branson Ultrasonics™, Danbury, CT, USA). The post-mitochondrial supernatant was obtained by centrifugation (10,000× *g*, 15 min, 4 °C), aliquoted and stored at −80 °C until the measurement of the biomarker activities. The assessed biomarkers were glutathione-*S*-transferase (GST), glutathione reductase (GR), glutathione peroxidase (GPx), total glutathione (TG), catalase (CAT), and acetylcholinesterase (AChE).

The enzymes activities were expressed per mg of protein content in the samples. Protein concentration was determined according to the Bradford method [22], adapted to the microplate format, measuring the absorbance at 595 nm and using bovine γ-globulin as a standard. The GST activity was determined through the method of [23], adapted for microplate [24], quantifying the conjugation of the substrate 1-chloro-2,4-dinitrobenzene (CDNB) with reduced glutathione (GSH). The absorbance was measured at 340 nm and GST activity calculated as nmol CDNB conjugate formed per minute per mg of protein (ε = 9.6 × 10^−3^ M^−1^ cm^−1^). The GR activity protocol was adapted from [25] into microplate format. The reaction medium contained potassium phosphate buffer 0.05 M with reduced nicotinamide-adenine dinucleotide phosphate (NADPH) 0.23 mM, oxidised glutathione (GSSG) 1 mM, and diethylenetriaminepentaacetic acid (DTPA) 0.45 mM. NADPH disappearance was measured at 340 nm and determined as mmol of oxidized NADPH (NADP^+^) produced per minute per mg of protein (ε = 6.22 × 10^3^ M^−1^ cm^−1^). GPx activity assay was adapted from [26,27]. The reaction medium was composed of potassium phosphate buffer 0.05 M, with sodium azide 1 mM, ethylenediaminetetraacetic acid (EDTA) 1 mM, reduced glutathione 4 mM, NADPH 0.8 mM, and glutathione reductase 1 U mL^−1^. The reaction, starting with the addition of hydrogen peroxide (H_2_O_2_) 0.16%, was measured for NADPH disappearance, at 340 nm, and the results were determined as mmol of oxidized NADP^+^ produced per minute per mg of protein (ε = 6.22 × 10^3^ M^−1^ cm^−1^). TG was assessed as described by [28], measuring GSH turnover with 5,5-dithio-bis-(2-nitrobenzoic acid) (DTNB), in the presence of GR. TG results were expressed as μmol of recycled GSH per minute per mg of protein. CAT was measured according to [29], following the change in absorbance at 240 nm caused by dismutation of H_2_O_2_, and activity was expressed in μmol of H_2_O_2_ consumed per minute per mg of protein (ε = 40 M^−1^ cm^−1^). AChE activity was determined by the Ellman’s method, where thiocholine is formed by the AChE present in the samples, which reacts with DTNB, resulting in the development of a yellow colour, which is measured at 414 nm and presented as nmol of thiocholine produced per minute per mg protein [30].

#### 2.3.3. Proteolytic Activity

*P. dicentrarchi* was exposed to a range of concentrations of *H. sabdariffa* extract (0.22–1.94 mg mL^−1^) in 24-well plates, for 2 h, at 20 °C ± 1 °C, in order to test for its proteolytic activity. Nine replicates were tested per each concentration, including the negative and solvent controls. After exposure, the cells were washed, suspended in potassium phosphate buffer (0.1 M, pH 7.4), homogenized, and centrifuged (10,000× *g*, 15 min, 4 °C), as described above, and stored at −80 °C until further analysis.

The proteolytic activity of the crude extract was evaluated through the use of the casein-fluorescein isothiocyanate (FITC-casein) assay [31]. *P. dicentrarchi* lysates and FITC-casein solution were loaded onto a 96-well plate and incubated at 37 °C for 60 min. The fluorescence levels were measured at 0 and 60 min of incubation, at 538 nm emission and 485 nm excitation. A standard curve was performed with a FITC isomer solution serially diluted (0.5–2.5 μM), in order to quantify FITC-casein disassembly into FITC and casein isomers in the presence of the crude extract. The activity of proteases was expressed as nmol min^−1^ mg prot^−1^.

#### 2.3.4. Expression Analysis of Protease-Encoding Genes by qRT-PCR

The targeted proteases represented some of the main types of enzymes found in *P. dicentrarchi*, which have also been associated with fish infection, namely, cathepsin cysteine proteases and leishmanolysins [3,5,8].

The parasites (5 × 10^6^ cells mL^−1^) were exposed to a minimum (0.14 mg mL^−1^) and a maximum (0.80 mg mL^−1^) concentration of hibiscus extract, plus the negative control (culture medium without plant extract added), in 12-well microplates containing a total assay volume of 1 mL. Four replicates were considered per treatment and the assay was carried out at 20 °C ± 1 °C, during 4 h. At the end of the exposure, the suspensions with parasites from each test condition/replicate were individually centrifuged at 700× *g* for 5 min and washed in PBS 1×. The pelleted parasites were subjected to total RNA extraction using the NZY Total RNA Isolation kit (Nzytech, Lisboa, Portugal), following the manufacturer’s instructions. The total RNA was quantified with Qubit^®^ RNA BR Assay Kit, and 1 μg was used as input to perform the cDNA synthesis with NZY First-Strand cDNA Synthesis Kit (Nzytech, Lisboa, Portugal) according to manufacturer indications. The cDNA was stored at −20 °C until further use, being quantified with Qubit^®^ ssDNA Assay kit (Alfagene, Lisboa, Portugal).

Towards the relative quantification of proteases’ gene expression, a reverse transcription (RT) real-time PCR was run with primers designed from the DNA sequences encoding cathepsin L-like cysteine protease, catepsin 90 protease, and leishmanolysin-family protein (Table 1) of *P. dicentrarchi*, obtained from Genbank. The primers were synthesized by IDT (Integrated DNA Technologies), being their amplification efficiency between 104.3 and 105.4% (cf. Table 1). The housekeeping gene used for normalizing the qRT-PCR results was β-tubulin. The qRT-PCR reaction contained 1× NZYSpeedy qPCR Green Master Mix (Nzytech, Lisboa, Portugal), 59 ng cDNA, 400 nM of each primer, and RNA-free ultrapure water up to a total reaction volume of 20 μL. Controls with no template were also performed for each primer pair and three biological replicates were performed for each assay. The PCR was performed in a CFX Connect Real-Time PCR Detection System (Bio-Rad Laboratories, Lisboa, Portugal), using the following cycling parameters: 95 °C for 2 min for enzyme activation, 40 cycles of denaturation at 95 °C for 5 s, and annealing at 60 °C for 15 s, and a final extension step at 60 °C for 15 s. In order to confirm the absence of unspecific products of amplification, a melting curve was done under the following conditions: 15 s at 95 °C, 15 s at 55 °C, heating gradually 0.2 °C per 5 s up to 95 °C. The qRT-PCR cycle threshold (Ct) values obtained were expressed as average and standard deviation. For each testing condition was computed the fold change in proteases gene expression following the application of method 2^−ΔΔCt^ [32], being:

ΔΔCt = [(Ct_(PDx)_ − Ct_(*ß*-*tubulin*)_)_extract_] − [(Ct_(PDx)_ − Ct_(*ß-tubulin*)_)negative control]

where, Ct is the cycle threshold, and PDx represents the target gene *x* (cf. Table 1).

### 2.4. Chemical Analysis of the Crude Extract

#### 2.4.1. Ultra-High-Performance Liquid Chromatography (UHPLC) Analysis

The separation of the compounds was carried out with a gradient elution program at a flow rate of 0.2 mL min^−1^, at 30 °C, by using a Hypersil Gold C18 column (100 × 2.1 mm; 1.9 µm) supplied by Thermo Fisher (Thermo Fisher Scientific, San Jose, CA, USA). The injection volume in the UHPLC system was 6 μL and the mobile phase consisted of formic acid 0.1% (A) and acetonitrile (B). The following linear gradient was applied: 0–14.7 min (5–40% B), 14.7–16.6 min (40–100% B), 16.6–18.8 min (100% B) 18.8–24 min (100–5%), followed by re-equilibration of the column for 10 min before the next run. Online detection was carried out in the diode array detector, at 280 nm, and UV spectra in a range of 190–700 nm were recorded.

#### 2.4.2. Electrospray Ionization Mass Spectrometric Detection (ESI–MS^n^) Analysis

The UHPLC was coupled to an LTQ XL Linear Ion Trap 2D mass spectrometer (Thermo Fisher Scientific, San Jose, CA, USA), equipped with an orthogonal electrospray ionization source operating in negative mode. The nitrogen sheath and auxiliary gas were 50 and 10 (arbitrary units), respectively. The spray voltage was 5 kV and the capillary temperature was 275 °C. The capillary and tune lens voltages were set at −28 V and −115 V, respectively. The data acquisition was carried out by using Xcalibur^®^ data system (Thermo Scientific, San Jose, CA, USA). The identification of compounds obtained by chemical analysis of the crude extract was carried out by comparing their chromatographic characteristics (e.g., retention time, UV-Vis maximum absorption (*λ_max_*, 190–700 nm), and mass spectrum (*m*/*z*)) with data available in the literature.

### 2.5. Statistical Analysis

Significant differences between the negative control and the solvent control or the extract concentrations were detected through the application of a one-way analysis of variance (one-way ANOVA), followed by the post-hoc multicomparison Dunnett’s or Tukey test (α = 0.05), for the following parameters: population growth rate, oxidative stress biomarkers, protease activity inhibition, differential expression of protease genes. No significant differences were determined between the negative and the solvent controls, thus, the latter was not plotted. The NOEC (no-observed effect concentration) and LOEC (low-observed effect concentration) values were determined for each parameter (except for gene expression outcomes). These analyses were performed using SigmaPlot 14.0. The IC_50_ (concentration inhibiting 50% of a biological parameter) values were determined by nonlinear regression for the population growth rate and proteases activity, using Graph Pad Prism 8.0 software (GraphPad Software Inc., Boston, MA, USA), being selected the nonlinear model that provided the best fit (higher R^2^ values) and lower-range confidence intervals.

## 3. Results

### 3.1. Effect of Hibiscus Extract on P. dicentrarchi Biological Responses

**Figure 1:**
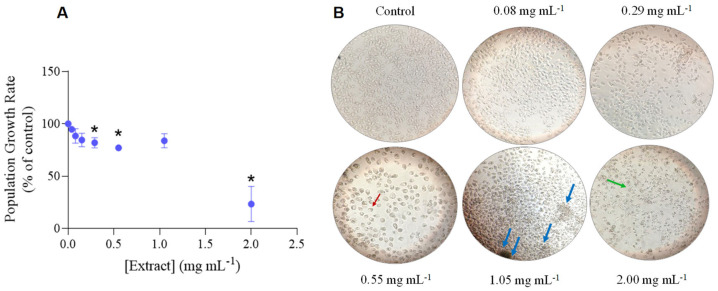
Effect of *Hibiscus sabdariffa* ethanol extract on *Philasterides dicentrarchi* proliferation. (**A**) Average growth rate of the ciliates under increasing concentrations of hibiscus crude extract ([Extract]). Results are expressed as a percentage of the negative control. The error lines correspond to the standard deviation. * Significantly different from the control (*p* < 0.05). (**B**) Photographs of *P. dicentrarchi* in the negative control and under some concentrations (in mg L^−1^) of *H. sabdariffa* extract, obtained in an inverted microscope with 50× or 100× (only for 0.55 mg mL^−1^ extract) magnification. The red arrow indicates a ciliate with shrunk cell content; the blue arrows highlight agglomerates of dead/disrupted cells; the green arrow points for disrupted cells.

The growth rate of *P. dicentrarchi* population had progressively decreased under increasing concentrations of *H. sabdrariffa* crude extract, being significantly inhibited at a LOEC of 0.29 mg mL^−1^ with an IC_50_ of 1.57 mg mL^−1^ (Figure 1A, Table 2). The steep reduction of the population growth rate under higher doses of the extract was associated with cell disruption and the appearance of aggregates of death cells, as microscopically observed (cf. Figure 1B).

**Figure 2:**
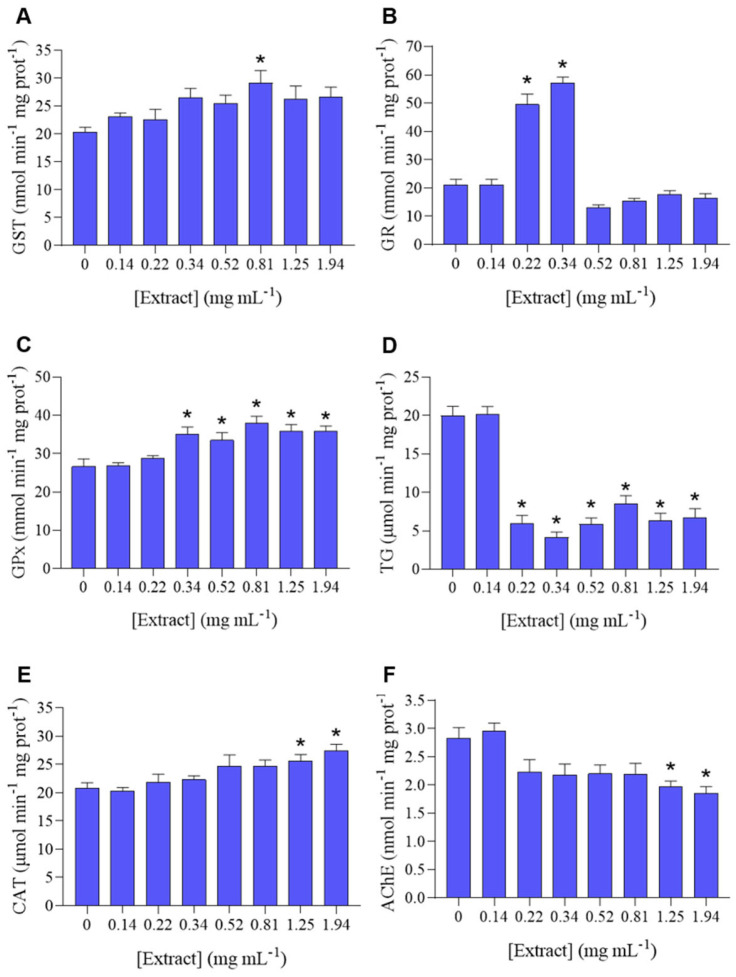
Oxidative stress biomarkers in *Philasterides dicentrarchi* exposed to increasing sub-lethal concentrations of *Hibiscus sabdariffa* crude extract ([Extract]). (**A**) Glutathione-*S*-transferase (GST); (**B**) Glutathione reductase (GR); (**C**) Glutathione peroxidase (GPx); (**D**) Total glutathione (TG); (**E**). Catalase (CAT); (**F**) Acetylcholinesterase (AChE). Results are expressed as mean ± standard error. * Significantly different from control (*p* < 0.05).

*P. dicentrarchi* was exposed to several sub-lethal concentrations of *H. sabdariffa* extract, to evaluate its effect on the ciliates’ redox balance by assaying biotransformation and antioxidant enzyme activities (GST, GR, GPx, TG, CAT), as well as the activity of AChE. A general increase in GST activity was observed in all concentrations, however, only statistically significant at 0.81 mg mL^−1^ (Figure 2A). There was a significant increase in GR activity at 0.22 and 0.34 mg mL^−1^, and the lowest concentration caused no effect (Figure 2B). GPx activity remained mostly unchanged at the lowest extract concentrations (0.14 and 0.22 mg mL^−1^), and significantly increased at 0.34, 0.52, 0.81, 1.25, 1.94 mg mL^−1^, comparatively to the control (Figure 2C). In terms of TG, there was a significant decrease in TG at concentrations between 0.22 and 1.94 mg mL^−1^ (Figure 2D). CAT (Figure 2E) exhibited a pattern of increase in activity along increasing concentrations of extract, with the two highest concentrations (1.25 and 1.94 mg mL^−1^) causing a significant increase comparatively to the control. AChE (Figure 2F) exhibited a pattern of decreasing activity in all concentrations (except the lowest), with a significant decrease in AChE activity on the two highest concentrations (1.25 and 1.94 mg mL^−1^).

**Figure 3:**
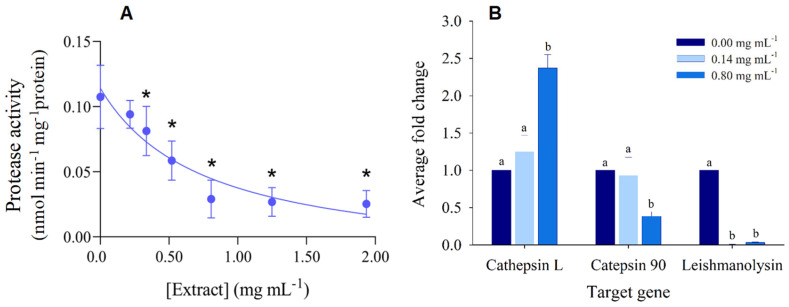
Effect of hibiscus crude extract on *Philasterides dicentrarchi* proteases. (**A**) Protease activity in the presence of increasing concentrations of *H. sabdariffa* crude extract. The results are expressed as average of protease activity (nmol min^−1^ mg protein^−1^). Error lines represent standard deviation. * Significantly different from the control, according to the Dunnett’s test (*p* < 0.05). (**B**) Differential expression (normalized relatively to the negative control and the reference gene *β-tubulin*) of protease-encoding genes under a low and a high concentration of the hibiscus extract. Error lines represent standard deviation. The letters above the bars indicate treatments or concentrations significantly different from each other for the respective target gene, according to the Tukey test (*p* < 0.05).

In what concerns the impact of hibiscus ethanolic extract on the protease activity of *P. dicentrarchi*, it was observed a significant inhibition under higher concentrations, allowing the estimation of a LOEC of 0.34 mg mL^−1^ and an IC_50_ of 0.76 mg mL^−1^ (Figure 3A, Table 2). For the evaluation of the differential expression of proteases genes, a preliminary step consisted on the analysis of melting curves for each sample and target gene, which confirmed that no unspecific products were formed during the PCR run. Hence, only one peak was observed at the melting temperature of the *β-tubulin* (housekeeping/reference gene) and target genes (Figure S1). Cathepsin L-like cysteine protease was significantly up-regulated under the higher concentration of the hibiscus ethanolic extract, whilst catepsin 90 was significantly downregulated (Figure 3B). In contrast, the leishmanolysin-family proteases were significantly down-regulated under both concentrations of the *H. sabdariffa* crude extract (Figure 3B).

### 3.2. Qualitative Chemical Analysis of Hibiscus Extract

**Figure 4:**
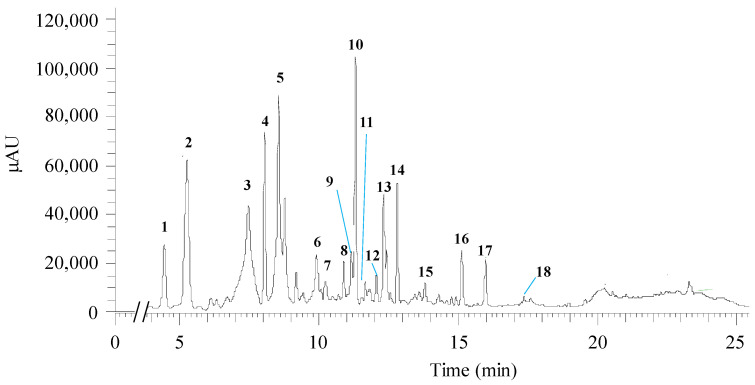
Chromatographic profile acquired from the qualitative analysis of *H. sabdariffa* crude ethanolic extract by UHPLC-ESI-MS^n^. The numbers highlight the base peaks analyzed in Table 3. AU—arbitrary units.

The chemical analysis of *H. sabdariffa* ethanol crude extract performed through UHPLC-ESI-MS^n^ allowed the identification of two organic acids and fifteen polyphenolic compounds. Among the latter were detected four phenolic acids, six flavonoids, and five anthocyanins. Table 3 lists the tentative identification of the compounds (peak numbered in the chromatogram in Figure 4) and the respective bioactivities reported in the literature.

More specifically, at 4.44 min, the ion found with *m*/*z* 189 could correspond to hibiscus acid, showing a *λ_max_* at 277 nm. A hibiscus acid derivative, hibiscus acid hydroxyethyldimethylesther, was also tentatively identified at 9.91 min. At 5.27 min, the *m*/*z* 353 (*λ_max_* = 324 nm) was possibly identified as 3-*O*-caffeoylquinic acid (3-CQA). Another chlorogenic acid was apparently found, 4-*O*-caffeoylquinic acid (4-CQA), with 8.04 min of retention time, *λ_max_* of 325 nm, and *m*/*z* 353. Cyanidin 3,5-*O*-diglucoside was tentatively identified in conjunction with 5-*O*-caffeoylquinic acid (5-CQA) (retention time: 10.22 min, *λ_max_* = 317 nm, *m*/*z*: 611, 353). 5-*O*-caffeoylshikimic acid (*m*/*z*: 335) and petunidin-3-*O*-glucoside (*m*/*z*: 479) are the proposed compounds at 10.89 min with *λ_max_* = 328 nm.

The 9th peak (Figure 4) was possibly assigned to the flavonoid quercetin-*O*-sambubioside (RT = 11.16 min, *λ_max_* = 354 nm, *m*/*z*: 595). At the retention time of 11.31 min, *λ_max_* 240 and 325 nm, and *m*/*z* 381 the 10th peak (Figure 4) could be associated with the formation of a complex of 5-CQA and formic acid, which was used in the mobile phase for compound elution. Quercetin derivatives could be identified, namely quercetin-3-*O*-rutinoside (retention time 11.66; *λ_max_* 256 and 352 nm; *m*/*z* 609) and quercetin 3-*O*-diglucoside (retention time 12.06 min; *λ_max_* 256 and 353 nm; *m*/*z* 463). Myricetin was the flavonoid proposed at 13.81 min (peak 15, Figure 4), with *λ_max_* 252 and 368 nm, *m*/*z* 317. At 15.13 min retention time, the peak could correspond to *N*-feruloyltyramide (*λ_max_* = 240, 291 and 317 nm, and *m*/*z*: 312). Quercetin or delphinidin were potentially assigned to the peak at a retention time of 15.97 min, with *λ_max_* 255 and 368 nm, and *m*/*z* 301; whereas delphinidin-3-*O*-sambubioside could be recovered at 17.36 min, *λ_max_* of 287 nm, and *m*/*z* 597.

Overall, among the identified phytochemicals some had previously shown anti-diabetic (hibiscus acid), antioxidant (3-CQA, 4-CQA, 5-CQA, 5-*O*-caffeoylshikimic acid, petunidin-3-*O*-glucoside, delphinidin-3-sambubioside, delphinidin-3-*O*-sambubioside, delphinidine, cyanidin 3,5-*O*-diglucoside, myricetin, and *N*-anthocyanins), antimicrobial (e.g., 3-CQA, 4-CQA, 5-CQA, quercetin diglucoside), anti-inflammatory (e.g., cyanidin 3,5-*O*-diglucoside, quercetin-3-*O*-diglucoside), and antiprotozoal (quercetin-3-*O*-rutinoside) activities (Table 3).

## 4. Discussion

Scuticociliate infections have become an extremely concerning issue in aquaculture units, particularly in turbot aquaculture, given the difficulties in mitigating the outbreaks and rapidly counteract the consequent impacts in the production levels. Thus, it is of paramount importance to optimize or create more sustainable and effective measures to control *P. dicentrarchi* infections, while promoting a safe and environmentally-protective aquaculture. In this context, plant extracts can potentially eliminate pathogens in fish aquaculture, due to their rich content in secondary and bioactive metabolites. On the other hand, the exploitation of plant-based disease control agents may be more cost-effective and environmentally beneficial, due to their faster biodegradability [54]. Based on these assumptions, the current study focused on bioprospecting *H. sabdariffa* crude extract and elucidate the qualitative chemical composition, which may sustain its potential antiprotozoal activity against *P. dicentrarchi*.

The results achieved showed that the hibiscus extract had significantly depleted *P. dicentrarchi* population growth rate (Figure 1A, Table 2) up to 76.7% inhibition at 2.00 mg mL^−1^, causing high mortality of the individuals under higher extract concentrations (Figure 1B). As a matter of fact, a decrease in the population growth rate of the ciliates is normally resulting from an impairment on their reproduction and survival rates [55]. The literature is rife of studies (cf. Table 4 for a summary of the most significant works published) proving the antimicrobial activity of *H. sabdariffa* extracts against Gram-negative and Gram-positive bacteria, and fungi, but the effect on protozoa has been barely evaluated (e.g., [12,15,18,19,20,36]).

Jabeur et al. [35,36] confirmed the antibacterial and antifungal activities of the hydroethanolic extract and infusion (Minimum Inhibitory Concentrations, MIC: 0.075–0.60 mg mL^−1^) of *H. sabdariffa* against a panel of bacteria and fungi species that represent a public health concern. Similarly, Youns et al. [20] determined that the methanol extract of *H. sabdariffa* exhibited an inhibitory activity against the growth of *E. coli*, *P. aeruginosa*, *K. pneumoniae*, *S. typhi*, *B. subtilis*, *and S. aureus* (MIC: 6.25–12.5 mg mL^−1^), having an effect equivalent or greater than 0.04 mg mL^−1^ gentamicin. In turn, the same authors observed that 0.5 mg mL^−1^ *H. sabdariffa* methanolic extract caused 72% mortality of the flagellated protozoa *G. lamblia*, after 72 h of exposure [20], thereby evidencing the antiprotozoal effect of hibiscus. Broadly, the LOEC value herein determined for the *P. dicentrarchi* population growth rate (0.29 mg mL^−1^) is within or one-to-two orders of magnitude below the MIC values estimated for bacteria and fungi exposed to extracts of hibiscus prepared with different solvents (Table 4). In fact, the polarity of the solvents used during the extraction procedure constrains the type and amount of the chemical compounds recovered [36]. Consequently, a certain variability on the bioactivity level of the hibiscus extracts is expected, depending on the selected solvent. In the current study was used ethanol since it is considered a green solvent, i.e., it is safe, non-toxic and presents a low environmental risk [56]. Moreover, ethanol has been proving enhanced effectiveness to extract compounds with antimicrobial activity, as compared to other solvents, such as methanol and acetone [57]. Besides solvent polarity, the species- and strains-specific sensitivity can also assume a wide range and trigger different profiles of biological responses, especially when the test organisms (prokaryotes vs. eukaryotes) present distinct physiological and metabolic complexities. In fact, according to Budiño et al. [2] different strains can co-exist in the same aquaculture unit, and these strains may present varying levels of infectivity and sensitivity to anti-protozoal agents. Notwithstanding, the outcome achieved regarding the inhibition of *P. dicentrarchi* population growth rate suggests that the *H. sabdariffa* extract may be a valuable alternative in the future to control its proliferation in fish farming.

Species across distinct *taxa* have developed antioxidant, detoxification, and repair systems to avoid the accumulation of oxidative damage, in which ROS are biologically relevant substances [58,59]. Although the redox system is highly conserved between species, there are significant differences among *taxa*. In bacteria and invertebrates, the oxidative stress mechanism is speculated to have a major role in the adaptive response to abiotic stressors. However, in protozoa, this mechanism is still mostly unknown [58]. It has been postulated that GST is responsible for the detoxification of xenobiotics, by directly catalyzing biotransformation reactions, besides having an important role in the cellular antioxidant defense [60]. In the present study, the *H. sabdariffa* ethanolic crude extract caused an overall increase in GST activity (Figure 2A), suggesting that this is a biotransformation mechanism activated by the testing compound within the organism.

GR is a highly specific enzyme responsible for reducing oxidized glutathione (GSSG) into two molecules of reduced glutathione (GSH), thereby maintaining the cellular redox balance by keeping the ratio of GSH to GSSG high [25]. In turn, GPx is responsible for catalyzing the reduction of H_2_O_2_, or organic hydroperoxides, into water or corresponding alcohols, using GSH as an electron donor, and oxidizing this molecule into GSSG [61]. GPx and GR maintain the glutathione cycle, as the continual conversion of GSH into GSSG (and vice-versa) protect cells from oxidative injury by removing free radicals produced in the metabolism of xenobiotics [62]. TG was used in the present study as an additional indicator to understand the way *P. dicentrarchi* manages the exposure to *H. sabdariffa* extract. Broadly, the tested extract caused a dose-dependent increase in GPx activity and a transient increase in the GR activity (Figure 2B–D), suggesting that *H. sabdariffa* extract caused oxidative stress in *P. dicentrarchi*, which triggered the antioxidant system. While the glutathione cycle is ubiquitous, there are organism-specific variations [63]. However, there is still little knowledge on how protozoal species regulate the GSH/GSSG ratio, despite the variability among protozoal species. For instance, apicomplexan protozoa have a well-developed glutathione system, while the amitochondrial protozoans *Entamoeba histolytica*, *Giardia*, and *Trichomonas* do not possess a glutathione metabolism [64]. *Plasmodium* species have a parasite-specific GR, but contain no true GPx, with the most GPx-like protein being thioredoxin peroxidase [65]. GR and GPx gene expression was identified in the ciliate *Euplotes crassus* [66], whereas GST and GPx were identified in the ciliate *Tetrahymena thermophila* [67]. Despite this knowledge for the mentioned parasites, there are currently no such studies unravelling the *P. dicentrarchi* glutathione metabolism.

CAT represents one of the first lines of antioxidant defense and an immediate protective response to ROS resulting from environmental stress. CAT catalyzes the conversion of H_2_O_2_ into water and molecular oxygen in cells exposed to environmental stress [68]. In this work, CAT exhibited a dose-dependent increase in activity, which was significantly promoted under the two highest concentrations (1.25 and 1.94 mg mL^−1^) (Figure 2E), confirming the activation of the antioxidant system as a result of the exposure to the hibiscus extract. A previous study showed that *P. dicentrarchi* antioxidant defense was triggered when subjected to natural polyphenolic compounds, however, this field remains vastly unexplored. The exposure of *P. dicentrarchi* to the natural polyphenol resveratrol caused dose-dependent CAT inhibition and GST induction, as well as a significant reduction in oxygen consumption and increased ROS production, which are indicative of oxidative stress and inability to eliminate ROS [69].

The cholinesterase activity of *P. dicentrarchi* was significantly decreased under 1.25 and 1.94 mg mL^−1^ *H. sabdariffa* ethanolic extract (Figure 2F). The activity of cholinesterases is usually considered a marker of neuronal function in ecotoxicity. However, the presence and function of cholinesterases in protozoa is barely understood [70], and no studies were found regarding *P. dicentrarchi* cholinesterases. It has been speculated that the fibrillar structure connecting the bases of cilia is a primitive conducting organ analogous to the nervous system. Thus, the presence of cholinesterase in ciliate protozoa could be correlated with motility, as well as reproduction [71], what could partly underpin the observed significant depletion of the population growth rate. The presence of the cholinergic system in *Colpoda* sp. seems to be connected to cell-to-cell interaction other than cell motility [72]. Moreover, AChE-like proteins have been detected in *E. crassus* and *Paramecium primaurelia* [72,73]. AChE expression has been found in *Trypanosoma evansi*, possibly with the purpose of regulating acetylcholine, similarly to mammals [74].

*P. dicentrarchi* secrete proteases, most predominantly cysteine proteases of the cathepsin L, B and D protease subfamilies, which mediate host entry, the spread of the infection, and the disruption or modification of the function of immune cells and molecules [3,5]. In particular, cysteine proteases can degrade haemoglobin, as well as type-I collagen, a protein component of the vertebrate tissues [3], thereby favoring the progressive digestion of fish tissues. Previous studies have reported a dose-dependent increase in the activity of the protease caspase-3-like in *P. dicentrarchi* incubated with turbot leucocytes [5], followed by the apoptosis of these immune cells. This outcome evidenced that proteases may be a tool for ciliates to evade the fish immune response and sustain their histophagous behavior. In the present study, the *H. sabdariffa* ethanolic extract caused a significant dose-dependent decrease in the activity of *P. dicentrarchi* proteases (Figure 3A, Table 2), reaching an inhibition of 73% at 0.81 mg mL^−1^ of extract.

In order to get a closer understanding of the influence of hibiscus extract on the protease activity inhibition, the expression of specific *P. dicentrarchi* protease genes was analyzed. Although the expression of cathepsin L-like cysteine protease was notably stimulated by the hibiscus extract, the catepsin 90 protease was significantly downregulated at 0.80 mg mL^−1^. Hence, a successful outcome was accomplished for catepsin 90, but not entirely to cathepsin L-like cysteine. Notwithstanding, it should be further analyzed the efficiency range of this extract towards the inhibition of this and other proteases, in tandem with the expression profiles along fish infection. In fact, despite the previously reported role of cathepsins in host infection by *P. dicentrarchi* [3,4,5], they were already found to be downregulated in ciliates infecting turbot [8] and the American lobster [75]. In opposition, leishmanolysins were found to be upregulated in ciliates isolated from infected turbot [8]. Leishmanolysins are important virulence-mediator metalloproteases originally found on the membrane of *Leishmania* bacterial cells [76]. Leishmanolysins (e.g., *gp63*) are able to destroy cells and molecules, such as molecules involved in the humoral immune response of the host [77], thereby helping the parasite to evade host immune system. In our study, the expression of these proteases was downregulated under both concentrations of the hibiscus ethanolic extract (Figure 3B). Therefore, the hibiscus extract can block this process, by enhance the susceptibility of *P. dicentrarchi* to the turbot immune system, hence reducing its infectivity.

Plants are rich in protease inhibitors that mediate defense mechanisms against predators and phytopathogens. Such protease inhibitors can also assume an active role as signaling molecules in several metabolic pathways [78]. Protease inhibitors have been previously identified in *H. sabdariffa* [79], namely roseltides, which are cysteine-rich knottin-type protease inhibitors [80,81]. Besides, protease inhibitors against angiotensin I converting enzyme have been also detected, such as flavones, elastase, trypsin, and alpha-chymotrypsin [82]. Considering that the use of protease inhibitors has been highlighted as a valuable alternative for the treatment of protozoan infections (e.g., [83]), the inhibitory effect of the hibiscus extract is significantly attractive to build-up sustainable measures for controlling the virulent action of *P. dicentrarchi*.

The overall antiprotozoal effect of the ethanolic extract of hibiscus calyces can be attributed to the presence of organic acids, phenolic acids, flavonoids, and anthocyanins with bioactive nature (Table 3). Considering that the produced extract is a complex mixture of such bioactive phytochemicals, a synergistic effect from their combined action is likely to further induce the observed damages in several biological responses of the ciliates.

Previous studies had reported the chemical composition of *H. sabdariffa* extracts and, despite some variations on the identified compounds and their concentrations, they are consensual in highlighting the abundance of organic acids and, particularly, of phenolic compounds (e.g., [14,15]). The ethanolic extract of *H. sabdariffa* analyzed by Borrás-Linares et al. [15] presented organic acids like hibiscus acid, hydroxycitric acid, citric acid, and protocatechuic acid, among which the hibiscus acid was also identified in our study. Hibiscus acids are derived from (2*S*,3*R*)-2-hydroxycitric acid, whose biosynthetic pathway has not yet been unraveled, since hibiscus acid(-derived) substances are not synthesized by all plants [84]. Notwithstanding, the hibiscus acid has been pointed out as a fulcral organic acid for the bioactivity of *H. sabdariffa* extract [14,15,36].

Within the phenolic compounds (i.e., phenolic acids, flavonoids, and anthocyanins), phenolic acids are usually the most represented in hibiscus extracts, with particular abundance of 3-CQA, 4-CQA, 5-CQA [14,15,36,85], which were tentatively assigned to several compound peaks recovered from our ethanolic crude extract (cf. Figure 4, Table 3). 5-CQA has been shown to have antimicrobial action against *E. coli*, *S. aureus*, *Enterococcus faecium*, *Proteus vulgaris*, *P. aeruginosa*, *K. pneumoniae* and *C. albicans* [44]. 4-CQA has strong antioxidant activity, as well as antimicrobial and anticancer activities [86].

Flavonoids are other class of phenolic compounds detected in the extract, being quercetin, quercetin-3-*O*-rutinoside, quercetin-*O*-sambubioside, quercetin-3-*O*-diglucoside, myricetin, and *N*-feruloyltyramide, some of the flavonoids likewise determined in related works (e.g., [15,33,35,36,39]). Quercetin-3-*O*-diglucoside is a naturally occurring form of the flavonol quercetin with anti-inflammatory activity [49,87], while quercetin-3-*O*-rutinoside is composed of quercetin and rutinose, having anti-allergenic, anti-inflammatory, antitumor, antibacterial, and antiprotozoal activities [50].

Anthocyanins have been frequently identified in extracts of *H. sabdariffa* and/or within the *Hibiscus* genus, being the major responsible compounds for the hibiscus pigment. The often detected anthocyanins are delphinidin-3-glucoside, cyanidin-3-glucoside, delphinidin-3-sambubioside and cyanidin-3-sambubioside [36,84,88,89,90,91]. Similarly, our ethanolic extract presented derivative compounds, such as cyaniding-3,5-*O*-diglucoside, petunidin-3-*O*-glucoside, delphinidin, and delphinidin-3-*O*-sambubioside (Table 3). The cyaniding-3,5-*O*-diglucoside has anti-inflammatory properties [92], whilst petunidin-3-*O*-glucoside and delphinidin have high antioxidant and/or antimicrobial activity [93,94].

Thus, the phytochemicals detected in *H. sabdariffa* ethanolic crude extract may explain its effects on *P. dicentrarchi* population growth rate, oxidative stress, neurotoxicity and proteases inhibition, due to their known antioxidant, antimicrobial, and, most importantly, antiprotozoal activity.

## 5. Conclusions

*H. sabdariffa* ethanolic crude extract was able to damage *P. dicentrarchi* at the molecular (biochemical), cellular (gene expression), individual (reproduction and mortality), and population (proliferation) level. The highest extract concentration caused a 76.7% decline in the population growth rate, which was linked to a severe cell disruption and mortality of the individuals. Moreover, it was demonstrated that sub-lethal concentrations of *H. sabdariffa* extract caused oxidative stress and cholinesterase inhibition in *P. dicentrarchi*, with expressive and significant shifts in the activity of the enzymes. These results are further validated by over 73% inhibition of the protozoal protease activity, and the expression inhibition of genes encoding proteases that mediate the parasites’ infectivity.

The mixture of organic acids and phenolic phytochemicals tentatively identified in *H. sabdariffa* ethanolic extract may trigger its antiprotozoal activity, making this extract efficacious towards the mitigation of *P. dicentrarchi* proliferation, impairment of its redox balance and general inhibition of crucial virulence factors. Hence, this extract shows favourable properties and should be further investigated to unravel its effect on farmed fish, as well as to evaluate its applicability in sustainable disease-control alternatives for intensive aquaculture settings engaged with high standards of quality, security and welfare in turbot production.

## Figures and Tables

**Table 1:** Proteases, primers, size of expected gene fragments and efficiency of primers in qRT-PCR.

Gene	Proteases	Origin, Accession Number	Primer Sets (5′ 3′)	Amplicon Size (bp)	Efficiency of Primers (%)
PD1	Cathepsin L-like cysteine protease	*Philasterides dicentrarchi*, AFF59216.1	F: TCTTGAGAGCTTCTGCTGCCAC	271	105.1
			R: TCTTGGATGTTTAATTCGGTGCTGT		
PD2	Catepsin 90 protease	*Philasterides dicentrarchi*, QBH22552.1	F: TAGCTTCAATTGCTTCTGGTAGTCTTG	227	105.3
			R: ATCCATGTTTATTCCACATAGTCCATTAC		
PD3	Leishmanolysin family protein	*Philasterides dicentrarchi*, QBH22559.1	F: TGTTTTAGAAGATTCTGGATTTTATG	331	105.4
			R: TATGTCAATTATATTACTGTAGAAGC		
*ß-tubulin*	-	*Philasterides dicentrarchi*, MH444695.1	F: GTATGATCATTGATAACGAAGCCCTCTACG	323	104.3

**Table 2:** Results of the one-way ANOVA analysis carried out to assess the effects of *Hibiscus sabdariffa* crude extract in *Philasterides dicentrarchi* population growth rate, biomarkers, and protease activity. The toxicity point estimates regarding NOEC, LOEC, and IC_50_ values are also presented (mg mL^−1^). GST: glutathione-*S*-transferase; GR: glutathione reductase; GPx: glutathione peroxidase; TG: total glutathione; CAT: catalase; AChE: acetylcholinesterase; PGR: population growth; PA: protease activity; DF: degrees of freedom; SS: sum of squares; MS: mean square; P: probability; NOEC: no-observed effect concentration; LOEC: low-observed effect concentration; IC_50_: concentration that inhibited a parameter in 50% of the population; CL: confidence limits of the IC_50_ value; R^2^: goodness-of-fit of the model adjusted to the experimental data for the derivation of the IC_50_ value; nd: not determined.

Parameter	One-Way ANOVA Outcome					Toxicity Point Estimates				
	DF	SS	MS	F	*p*	NOEC	LOEC	IC_50_	CL	R^2^
PGR	7	11,841.83	1691.69	30.413	<0.001	0.15	0.29	1.57	1.09–2.31	0.67
GST	7	444.035	63.434	2.429	0.029	0.52	0.81	nd	nd	nd
GR	7	15,743.5	2249.071	67.499	<0.001	0.14	0.22	nd	nd	nd
GPx	7	1184.466	169.209	7.289	<0.001	0.22	0.34	nd	nd	nd
TG	7	1920.084	274.298	29.331	<0.001	0.14	0.22	nd	nd	nd
CAT	7	368.225	52.604	4.403	<0.001	0.81	1.25	nd	nd	nd
AChE	7	8.675	1.239	5.07	<0.001	0.81	1.25	nd	nd	nd
PA	6	0.0548	0.00913	37.99	<0.001	0.22	0.34	0.76	0.45–1.45	0.76

**Table 3:** *Hibiscus sabdariffa* crude extract compounds tentatively identified by electrospray ionisation mass spectrometry (ESI-MS; [M–H]^−^), and their respective bioactive function. RT: retention time; *λ_max_*: maximum absorbance wavelengths in the UV-VIS spectrum region; nd: not determined. 5-CQA: 5-*O*-caffeoylquinic acid; FA: formic acid.

Peak #	RT (min)	*λ*_max_	[M-H]^−^ (*m/z*)	MS^2^ Fragments (*m/z*)	Proposed Compound	Chemical Class	References	Bioactivity	References
**1**	4.44	277	189	[189] 127	hibiscus acid	Organic acids	[33,34]	Anti-diabetic	[14]
**2**	5.27	324	353	[353] 191; 179	3-*O*-caffeoylquinic acid	Phenolic acid (polyphenol)	[35,36]	Antioxidant, antibacterial, anticancer, antihistamine	[37,38]
**3**	7.46	281; 523	595	[595] 355; 300	delphinidin-3-sambubioside	Anthocyanin (polyphenol)	[39]	Antioxidant, antibacterial, antiviral, anthelmintic	[14,40,41]
**4**	8.04	325	353	[353] 191; 173	4-*O*-caffeoylquinic acid	Phenolic acid (polyphenol)	[35]	Antioxidant, antibacterial, anticancer, antihistamine	[37,38]
**5**	8.54	293; 325	353; 398.7	nd	Unknown	nd	nd	nd	nd
**6**	9.91	276; 528	263	[263] 217	hibiscus acid hydroxyethyldimethylesther	Organic acids	[15]	nd	nd
**7**	10.22	317	611	[611]	cyanidin 3,5-*O*-diglucoside	Anthocyanin (polyphenol)	[42]	Antioxidant, antibacterial, antiviral, anthelmintic	[40,41]
			353	[353] 191	5-*O*-caffeoylquinic acid (5-CQA)	Phenolic acid (polyphenol)	[33,35,43]	Antioxidant, antibacterial, anticancer, antihistamine	[37,38]
**8**	10.89	328	335	[335] 161; 135	5-*O*-caffeoylshikimic acid	Phenolic acid (polyphenol)	[15,33,43]	Potentially antioxidant and antimicrobial	[44]
			479	[479] 317	petunidin-3-*O*-glucoside	Anthocyanin (polyphenol)	[45,46]	Antioxidant	[47]
**9**	11.16	354	595	[595] 299.9	quercetin-*O*-sambubioside	Flavonoid (polyphenol)	[33]	Antibacterial, antiviral, anti-inflammatory	[48]
**10**	11.31	240; 325	381	[381] 161	5-CQA:formic acid (FA) complex	Phenolic acid (polyphenol) + organic acid	Based in 5-CQA *m/z* [33]	nd	nd
					(335 (5-CQA) + 46 (FA) = 381)				
**11**	11.66	256; 352	609	[609] 301	quercetin-3-*O*-rutinoside	Flavonoid (polyphenol)	[33]	Antibacterial, antitumoral, anti-inflammatory, anti-allergenic, antiviral, vasoactive, antiprotozoal	[49]
**12**	12.06	256; 353	463	[463] 301	quercetin-3-*O*-diglucoside	Flavonoid (polyphenol)	[33]	Antibacterial, antiviral, anti-inflammatory	[48]
**13**	12.33	239; 326	381	[381] 161; 135	Unknown	nd	nd	nd	nd
**14**	12.81	242; 327	381	[381] 179; 191; 135	Unkown				
**15**	13.81	252; 368	317	[317] 165	myricetin	Flavonoid (polyphenol)	[39]	Antioxidant, antimicrobial	[50]
**16**	15.13	240; 291; 317	312	[312] 297; 178; 135	*N*-feruloyltyramide	Flavonoid (polyphenol)	[33,43]	Antioxidant, antimicrobial, anticancer	[51]
**17**	15.97	255; 368	301	[301] 179; 151	quercetin or delphinidin	Flavonoid or Anthocyanin (polyphenol)	[33,43,46,52]	Antioxidant, anti-inflammatory, antibacterial, antiviral	[48,53]
**18**	17.36	287	597	[597] 579	delphinidin-3-*O*-sambubioside	Anthocyanin (polyphenol)	[34,36,42]	Antioxidant, antibacterial, antiviral, anthelmintic	[14,40,41]

**Table 4:** Summary of studies assessing effects of plant-based products on the growth and mortality of pathogenic microorganisms. LOEC: low observed effect concentration; MIC: minimum inhibitory concentration (in growth assays with bacteria); * MIC80 (minimum inhibitory concentration causing 80% growth inhibition).

Extract	Group of Organisms	Pathogen Species	Parameter	Exposure Time (h)	LOEC or MIC (mg mL^−1^)	Reference
			(In Vitro Assays)			
5-*O*-Caffeoylquinic acid	Bacteria	*Staphylococcus aureus*	Growth inihibition	48	10 *	[44]
		*Enterococcus faecium*			10 *	
		*Escherichia coli*			10 *	
		*Proteus vulgaris*			10 *	
		*Pseudomonas aeruginosa*			10 *	
		*Klebsiella pneumoniae*			5 *	
	Fungi	*Candida albicans*			10 *	
*H. sabdariffa* hydroethanolic extract	Bacteria	*Staphylococcus aureus*	Growth inhibition	24	0.15–0.45	[35,36]
		*Bacillus cereus*			0.15	
		*Micrococcus flavus*			0.20–0.45	
		*Listeria monocytogenes*			0.15–0.45	
		*Escherichia coli*			0.20	
		*Enterobacter cloacae*			0.15–0.3	
		*Salmonella typhimirium*			0.15–0.45	
		*Pseudomonas aeruginosa*			0.15–0.45	
	Fungi	*Aspergillus fumigatus*	Growth inhibition	72	0.30–0.45	
		*Aspergillus versicolor*			0.10	
		*Aspergillus ochraceus*			0.15	
		*Aspergillus niger*			0.30–0.60	
		*Penicillium ochrochloron*			0.20–0.30	
		*Penicillium verrucosum* var. *cyclopium*			0.20–0.30	
		*Trichoderma viride*			0.075	
*H. sabdariffa* infusion	Bacteria	*Staphylococcus aureus*	Growth inhibition	24	0.20–0.30	
		*Bacillus cereus*			0.10	
		*Micrococcus flavus*			0.30	
		*Listeria monocytogenes*			0.20–0.30	
		*Escherichia coli*			0.20	
		*Enterobacter cloacae*			0.30–0.15	
		*Salmonella typhimirium*			0.20–0.45	
		*Pseudomonas aeruginosa*			0.20–0.30	
	Fungi	*Aspergillus fumigatus*	Growth inhibition	72	0.30–0.60	
		*Aspergillus versicolor*			0.15–0.30	
		*Aspergillus ochraceus*			0.04	
		*Aspergillus niger*			0.30–0.60	
		*Penicillium ochrochloron*			0.20–0.30	
		*Penicillium verrucosum* var. *cyclopium*			0.30–0.60	
		*Trichoderma viride*			0.075–0.30	
*H. sabdariffa* methanolic extract	Bacteria	*Escherichia coli*	Growth inhibition	24	12.5	[20]
		*Pseudomonas aeruginosa*			12.5	
		*Klebsiella pneumoniae*			12.5	
		*Salmonella typhi*			12.5	
		*Bacillus subtilis*			6.25	
		*Staphylococcus aureus*			6.25	
	Protozoa (ciliate)	*Giardia lamblia*	Mortality	24, 48, 72, 96	0.125	
*H. sabdariffa* ethanolic extract	Protozoa (ciliate)	*Philasterides dicentrarchi*	PGR	96	0.29	This study
			Proteases activity	4	0.34	

## Data Availability

Data is contained within the article or supplementary material.

## Supplementary Materials

The following supporting information can be downloaded at: https://www.mdpi.com/article/10.3390/biology12070912/s1, Figure S1: Melt curves obtained in qRT-PCR runs for *β-tubulin* (reference gene), and cathepsin L-like cysteine, catepsin 90 and leishmanolysin protease genes.
