# Supplementary material for: In Vitro Antiprotozoal Activity of Hibiscus sabdariffa Extract against a Ciliate Causing High Mortalities in Turbot Aquaculture
Source: Biology (Basel). 2023 Jun 26;12(7):912. doi: 10.3390/biology12070912 (PMC10376481; doi:10.3390/biology12070912)
Supplement: Supplementary file 1 [file biology-12-00912-s001.zip › biology-2430520-supplementary.pdf]

Supplementary material

In vitro antiprotozoal activity of *Hibiscus sabdariffa* extract against a ciliate causing high mortalities in turbot aquaculture

Ana Carvalho[1], Inês Domingues [1], Carla Carvalho [1], Artur M.S. Silva [2], Amadeu M.V.M. Soares [1], Catarina R. Marques [1,*]

[1] Centre of Marine and Environmental Studies (CESAM), Department of Biology, University of Aveiro, Santiago University Campus, 3810-193 Aveiro, Portugal

[2] Laboratório Associado para a Química Verde (LAQV) - REQUIMTE, Department of Chemistry, University of Aveiro, Santiago University Campus, 3810-193 Aveiro, Portugal

* Correspondence: crmarques@ua.pt

Supplementary Figure

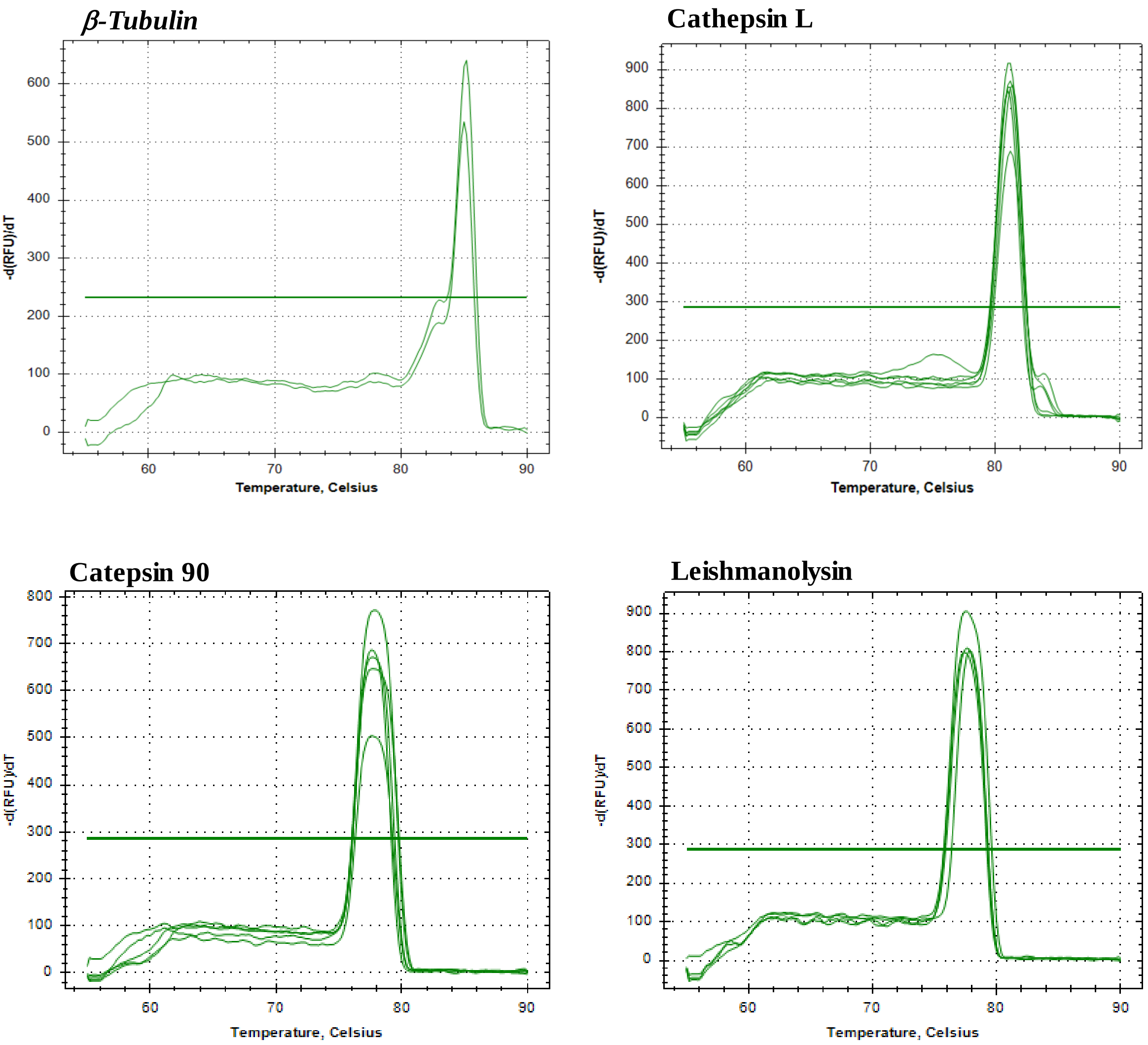

Figure S1: Melt curves obtained in RT-PCR runs for *β-tubulin* (reference gene), and cathepsin L-like cysteine, catepsin 90 and leishmanolysin protease genes.
